# The complete chloroplast genome of *Urtica angustifolia* Fisch. ex Hornem. (Urticaceae), an important kind of traditional Chinese medicine in China

**DOI:** 10.1080/23802359.2022.2057246

**Published:** 2023-01-15

**Authors:** Mu Liu, Jinsen Lu, Baoyong Li, Lvshui Zhang

**Affiliations:** College of Landscape Architecture and Arts, Jiangxi Agricultural University, Nanchang, China

**Keywords:** *Urtica angustifolia*, phylogenetic relationship, chloroplast genome

## Abstract

*Urtica angustifolia* Fisch. ex Hornem. is an important Chinese medicine. Here, the complete chloroplast genome of *U. angustifolia* was assembled and characterized. The length of the chloroplast genome was 146,679 bp with the typical quadripartite structure, containing two inverted repeats (IRs) of 24,595 bp separated by a large single-copy (LSC) region of 79,820 bp and a small single-copy (SSC) region of 17,669 bp. The whole chloroplast genome of *U. angustifolia* contains 111 genes, including 77 protein-coding genes, 30 tRNA genes, and 4 rRNA genes. Nucleotide variability analysis identified three hotspot regions (*trnK-rps16*, *ndhF-rps32*, and *ycf1b*) for genomic divergence and 52 simple sequence repeats. Phylogenetic analysis based on the complete chloroplast genomes exhibited that *U. angustifolia* formed a clade with *Urtica lobatifolia* and *Hesperocnide tenella*.

*Urtica angustifolia* Fisch. ex Hornem., belonging to Urticaceae, is mainly distributed in northern China, eastern Siberia, Russia, Mongolia, Korea and Japan. *Urtica angustifolia* is a traditional medicinal material (Zhang and Li 2012). However, the genetic information was less available. The chloroplast genome sequences provide an effective genetic resource for resolving complex evolutionary relationships, assessing population genetics, and identifying species (Dong, Liu, et al. 2021; Dong, Sun, et al. 2021; Wang, et al. 2021; Dong, Liu, et al. 2022). In the current study, we sequenced the complete chloroplast genome of *U. angustifolia* using Illumina Hiseq X ten platform.

Fresh and healthy herb material of *U. angustifolia* was collected from Maorshan mountain, Shangzhi, Heilongjiang, China (45°17′55″–127°31′49″). The collection of plant material was in accordance with local regulations and obtain the permission of local authorities. The voucher specimen was deposited at the herbarium of Jiangxi Agricultural University under the voucher number of ENC850487 (Mu Liu, aawolongaa@163.com). The total genomic DNA was extracted from the fresh leaves with the modified CTAB method (Li et al. 2013). Genomic DNA was fragmented to construct a shotgun library with an insert size of 350 bp. The library was sequenced using the Illumina HiSeq X-ten platform and approximately 4 Gb data was generated from the sequencing library. Raw data were qualified by using Trimmomatic (Bolger et al. 2014) and the chloroplast genome was assembled with GetOrganelle (Jin et al. 2019). The complete chloroplast genome of *U. angustifolia* was annotated with Plann (Huang and Cronk 2015) using *U. lobatifolia* (Urticaceae, GenBank accession number: MW246155) as a reference. The annotated chloroplast genome of *U. angustifolia* has been deposited into GenBank with the accession number of MZ145046.

The length of the *U. angustifolia* chloroplast genome is 146,679 bp including two inverted repeats (IR, 24,595 bp), a large single-copy region (LSC, 79,820 bp) and a small single-copy region (SSC, 17,669 bp). The GC content of the genome is 36.6%. The *U. angustifolia* chloroplast genome is predicted to contain 111 unique genes, including 77 protein-coding genes, 30 tRNA genes and 4 rRNA genes. Among these genes, 12 genes (a*tpF, ndhA, ndhB, petB, petD, rpoc1, rpl16, trnA-UGC, trnI-GAU, trnK-UUU, trnL-UAA,* and *trnV-UAC*) exhibit one intron and two genes (*clpP* and *ycf3*) contain two introns.

The Perl script MISA was used to identify microsatellites with the minimum numbers of repeats set to 10, 5, 4, 3, 3, and 3 for mono-, di-, tri-, tetra-, penta-, and hexanucleotides, respectively. The total number of SSRs identified in *U. angustifolia* chloroplast genome was 52. We calculated the nucleotide diversity (PI) using sliding window method (window size: 800 bp and step size: 200 bp) to identify the mutation hotspots in the chloroplast genome alignments of three *Urtica* samples and *Hesperocnide tenella*. Three variable regions (*trnK-rps16*, *ndhF-rps32*, and *ycf1b*) were found to be more variable with Pi values >0.05. We also identified 403 indels in the four aligned chloroplast genomes.

To resolve the phylogenetic position of *U. angustifolia*, a total of 36 chloroplast genome sequences (one outgroup from Moraceae) were downloaded from the NCBI database. Sequences were aligned using MAFFT (Katoh and Standley 2013) and the ambiguous alignment regions were trimmed using Gblocks 0.91 b (Castresana 2002). The maximum-likelihood (ML) phylogenetic tree was reconstructed using RAxML (Stamatakis 2014). ML analysis was performed using RAxML with 500 replications under the GTR + G model. The result showed that Urticaceae was divided into two clades and *U. angustifolia* formed a clade with *Urtica lobatifolia* and *Hesperocnide tenella* (Figure 1). This result is consistent with the phylogenetic relationship based on several chloroplast genes, which also supported *Hesperocnide tenella* was within the genera *Urtica* (Wu et al. 2013, 2018). Our study will provide valuable insight into conservation genetics, taxonomy, and evolutionary histories for this particular species.

**Figure 1.**
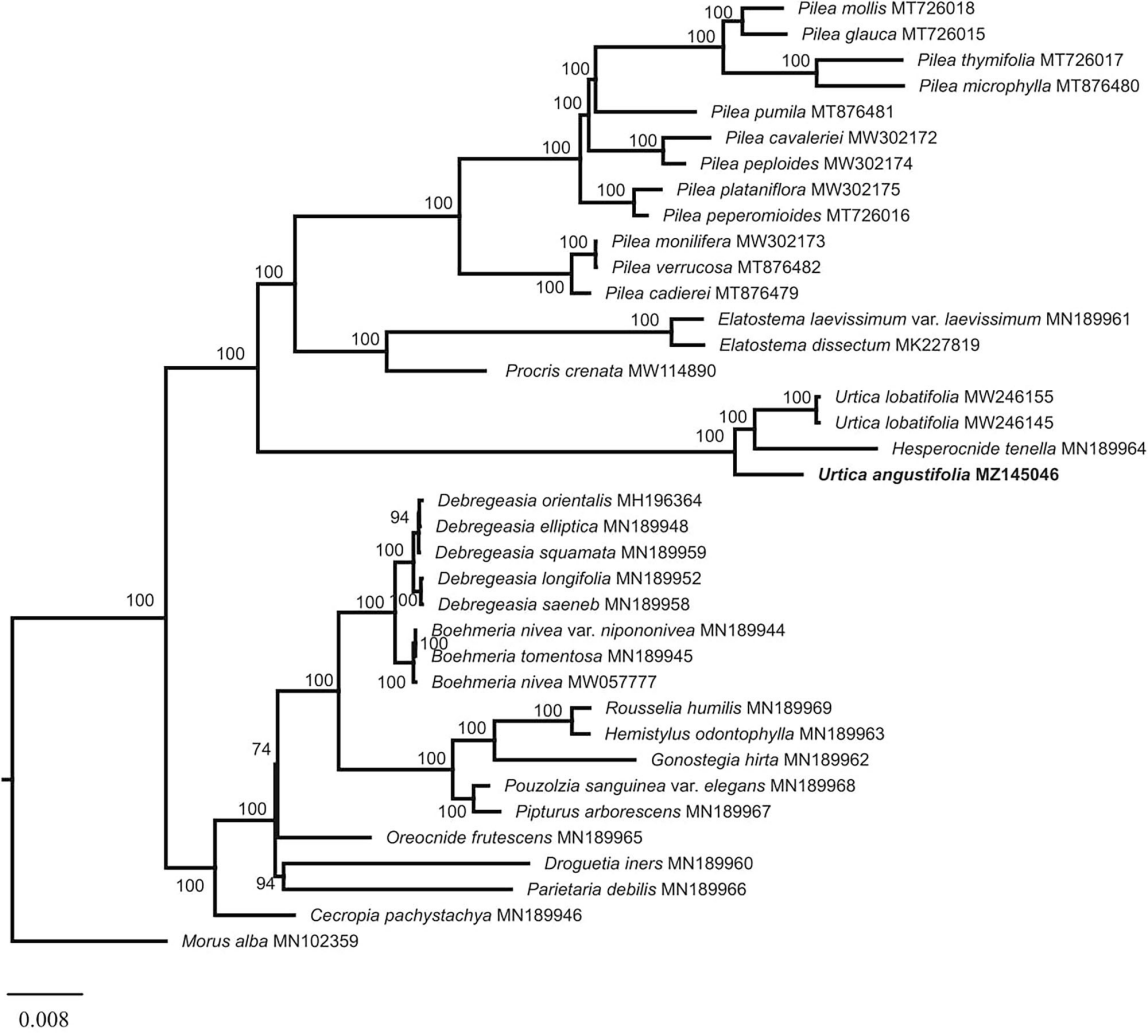
Phylogenetic tree of Urticaceae based on 37 complete chloroplast genome sequences. ML topology shown with ML bootstrap support value at each node.

## Data Availability

The genome sequence data that support the findings of this study are openly available in GenBank of NCBI at (https://www.ncbi.nlm.nih.gov/) under the accession no. MZ145046. The associated BioProject, SRA, and Bio-Sample numbers are PRJNA749813, SRR15253676, and SAMN20422813, respectively.
